# Supplementary material for: Three host peculiarities of a cycloalkane-based micelle toward large metal-complex guests
Source: Nat Commun. 2020 Nov 27;11:6061. doi: 10.1038/s41467-020-19886-4 (PMC7695700; doi:10.1038/s41467-020-19886-4)
Supplement: Supplementary file 1 — Supplementary Information [file 41467_2020_19886_MOESM1_ESM.pdf]

Supplementary Information

Three host peculiarities of a cycloalkane-based micelle toward large metal-complex guests

Hanafusa et al.

Supplementary Figures

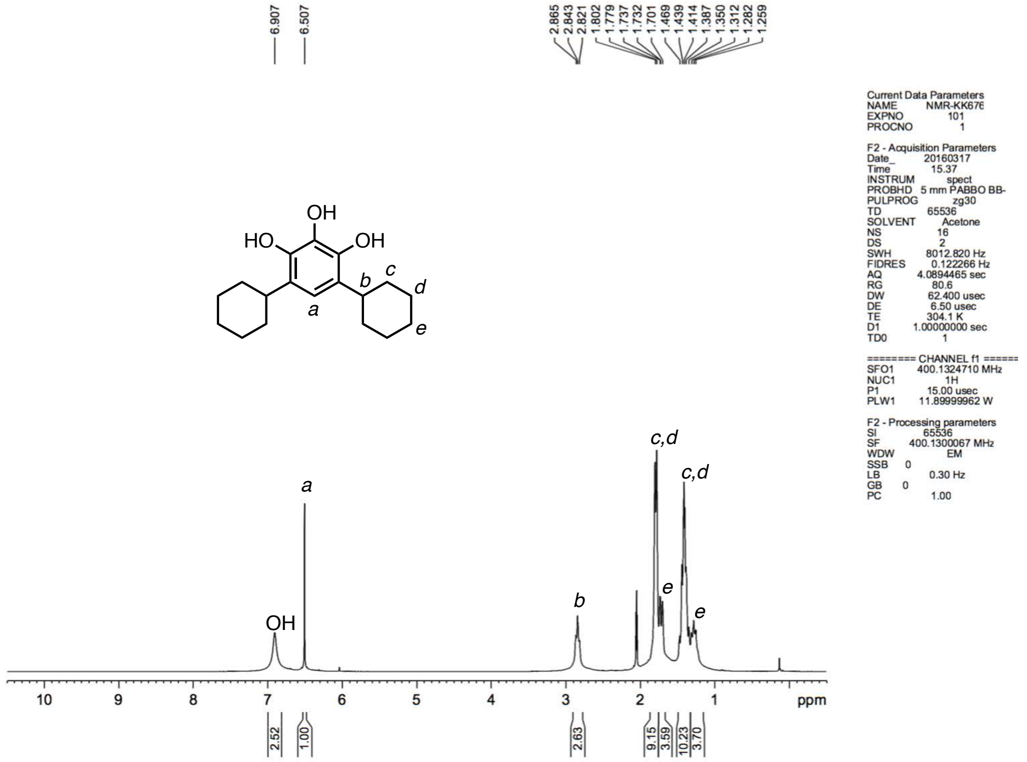

Supplementary Fig. 1 ^{1}H NMR spectrum (400 MHz, acetone-d_6, room temperature) of 1,5-dicyclohexyl-2,3,4-trihydroxybenzene.

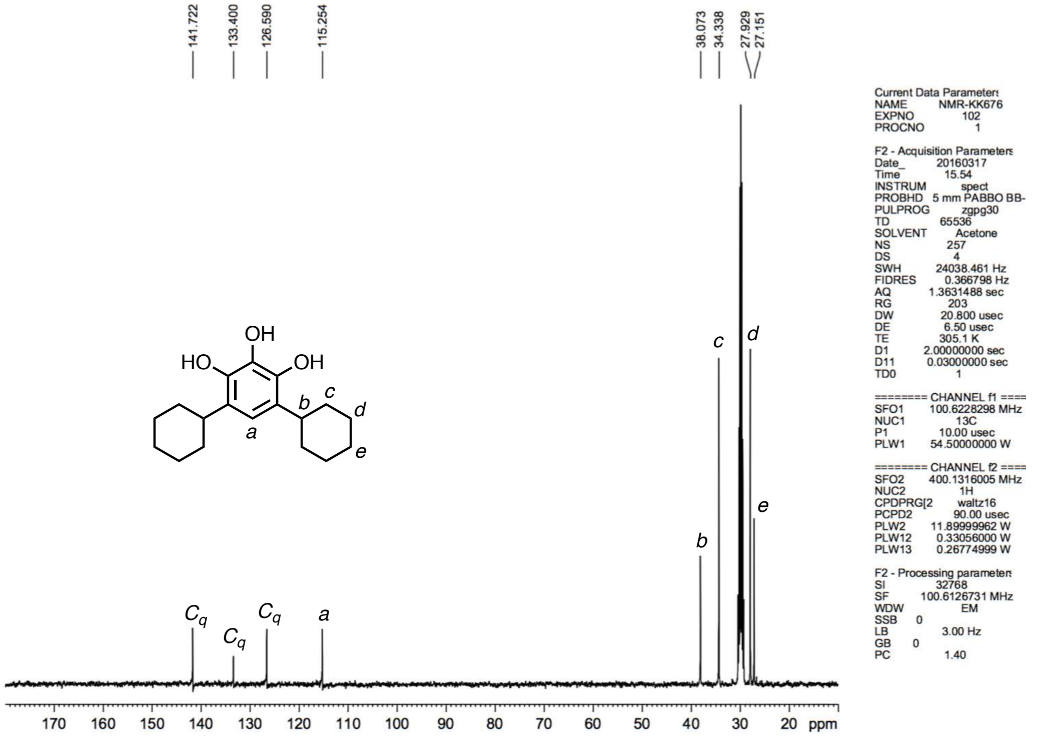

Supplementary Fig. 2 ^{13}C NMR spectrum (100 MHz, acetone-d_6, room temperature) of 1,5-dicyclohexyl-2,3,4-trihydroxybenzene.

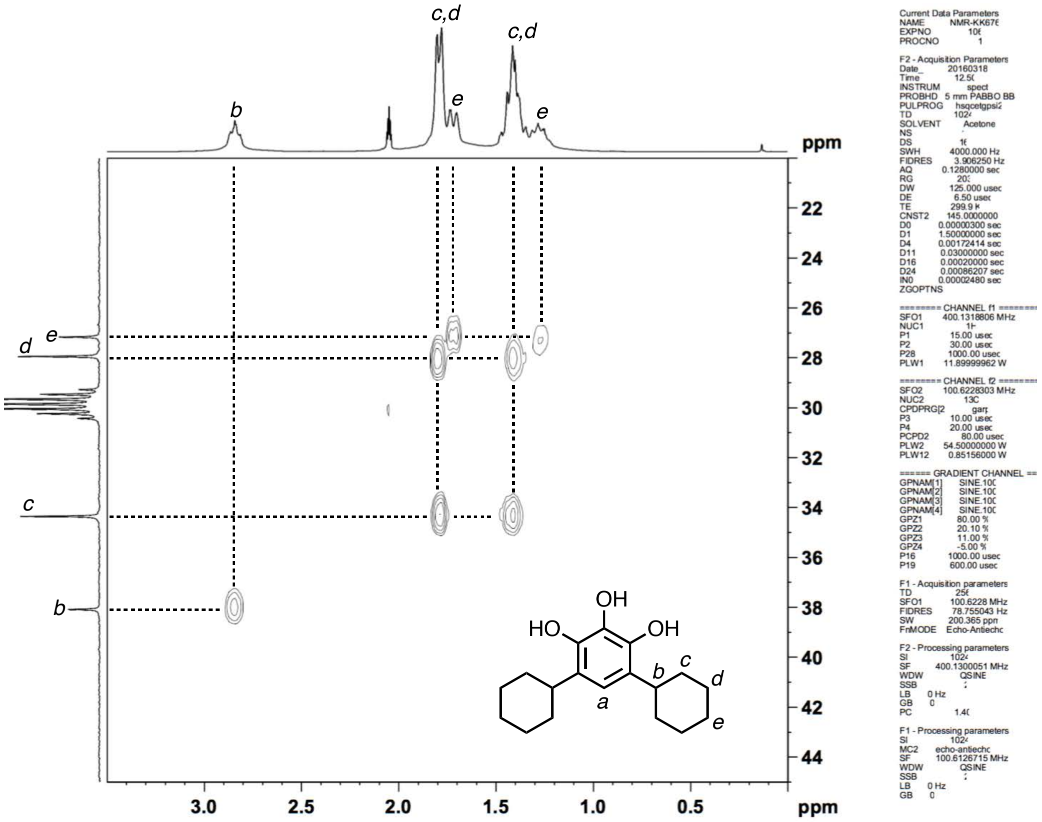

Supplementary Fig. 3 HSQC spectrum (400 MHz, acetone-d_6, room temperature) of 1,5-dicyclohexyl-2,3,4-trihydroxybenzene.

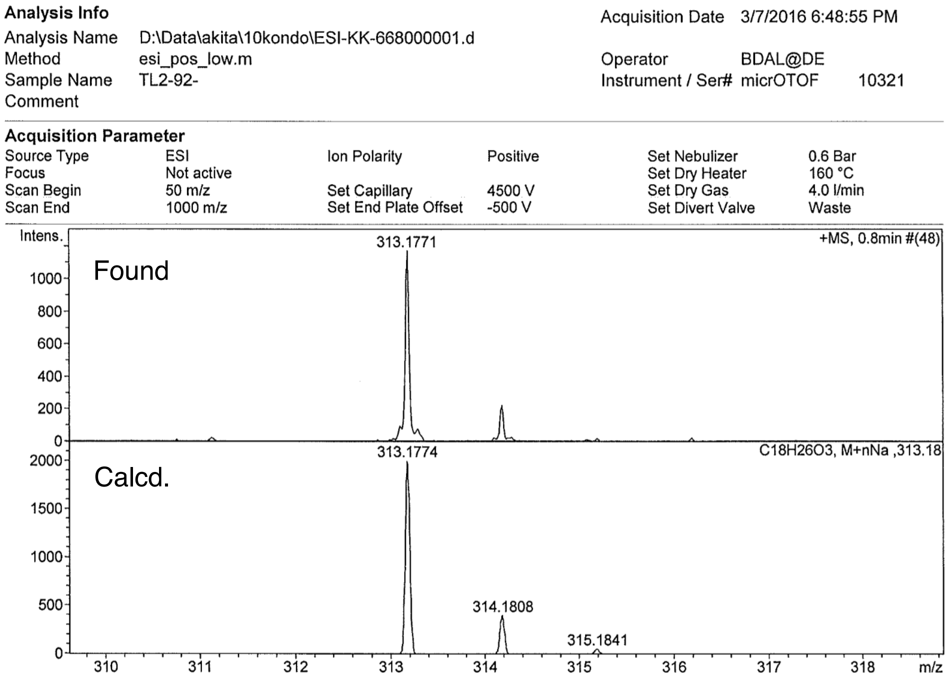

Supplementary Fig. 4 HR MS spectrum (ESI, CH_3OH, room temperature) of 1,5-dicyclohexyl-2,3,4-trihydroxybenzene.

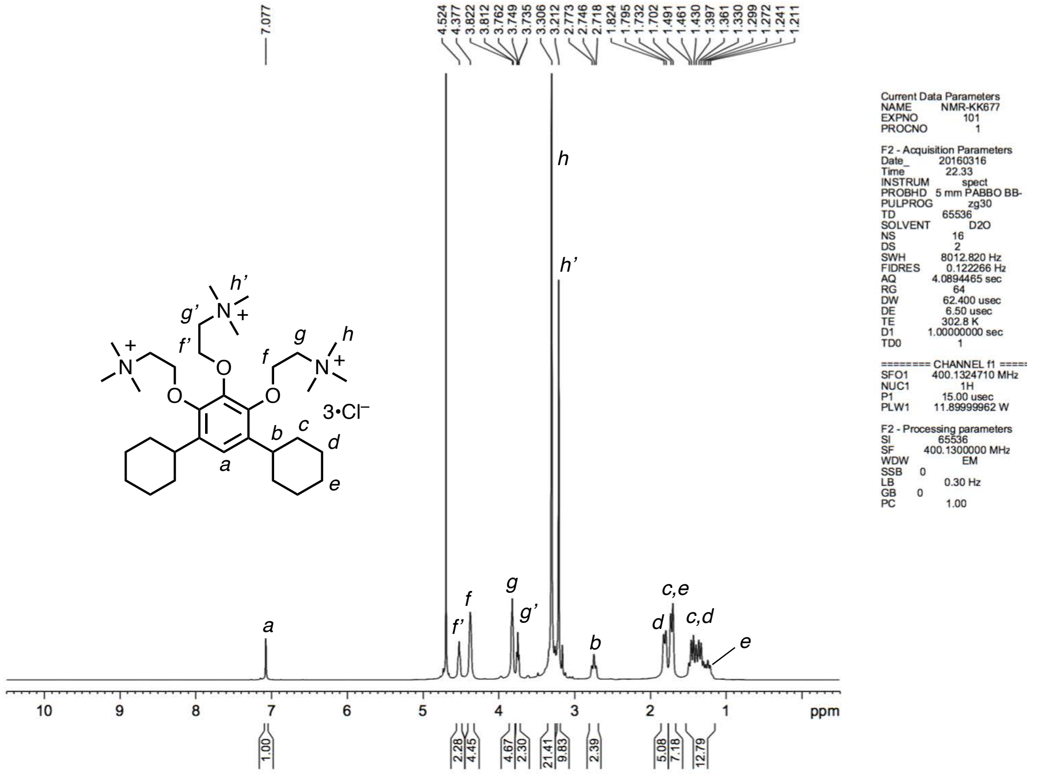

Supplementary Fig. 5 ^{1}H NMR spectrum (400 MHz, D_2O, ~2 mM, room temperature) of **CHA**.

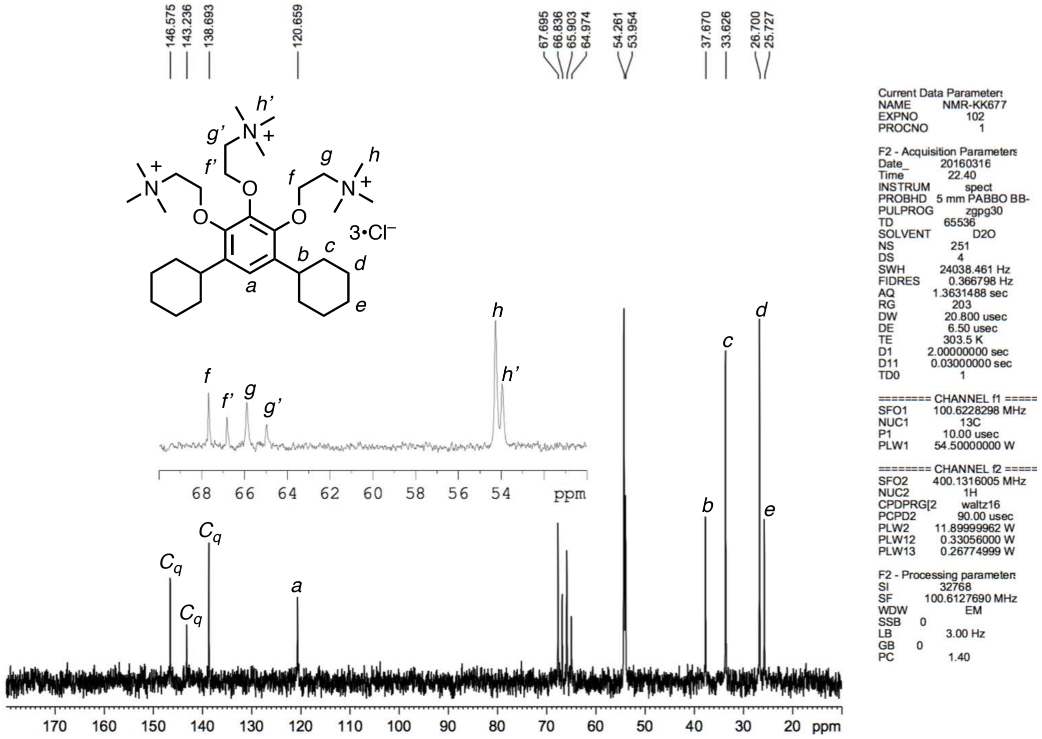

Supplementary Fig. 6 ^{13}C NMR spectrum (100 MHz, D_2O, ~2 mM, room temperature) of **CHA**.

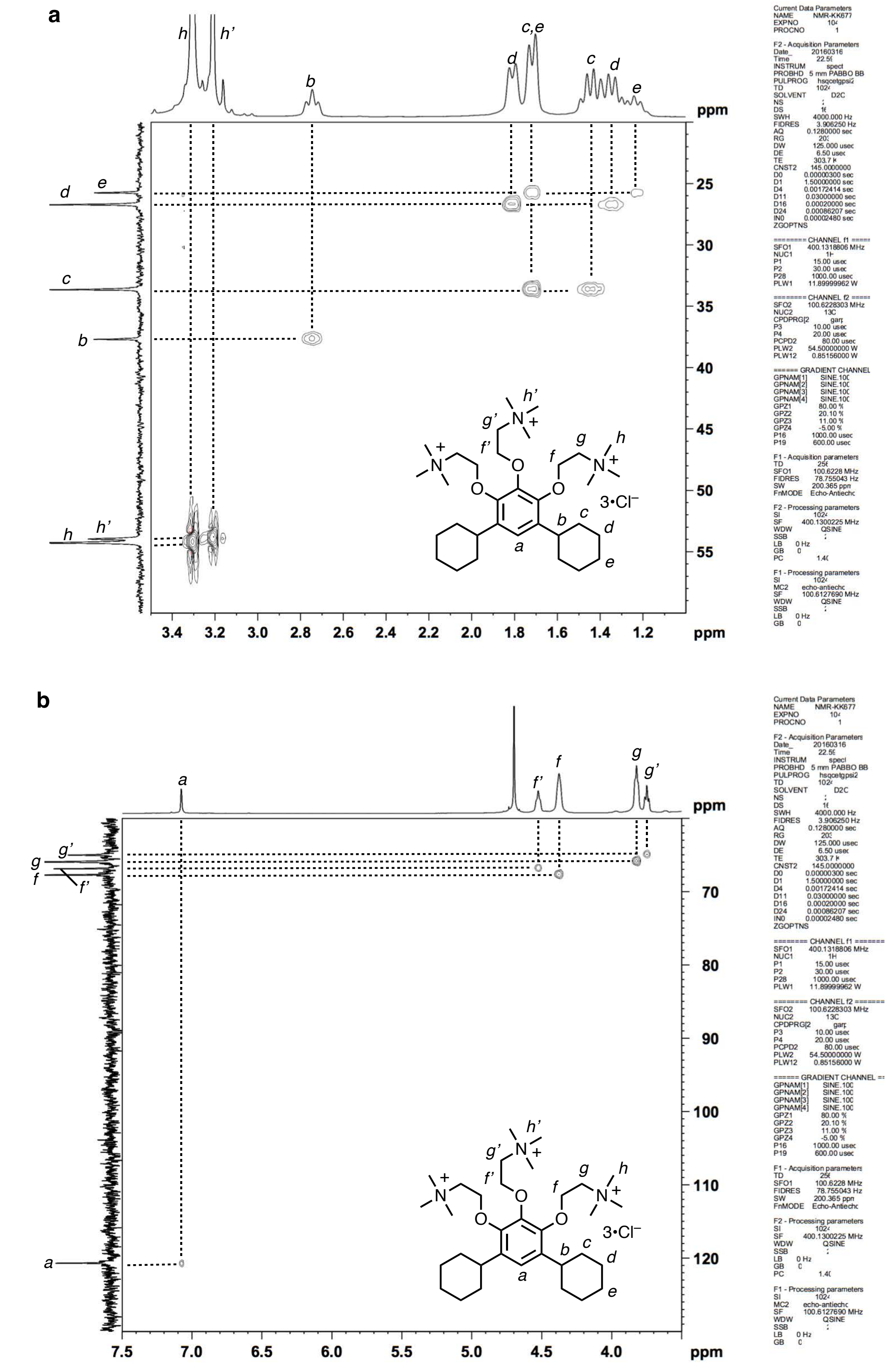

Supplementary Fig. 7 HSQC spectra (400 MHz, D_2O, ~2 mM, room temperature) of **CHA**.

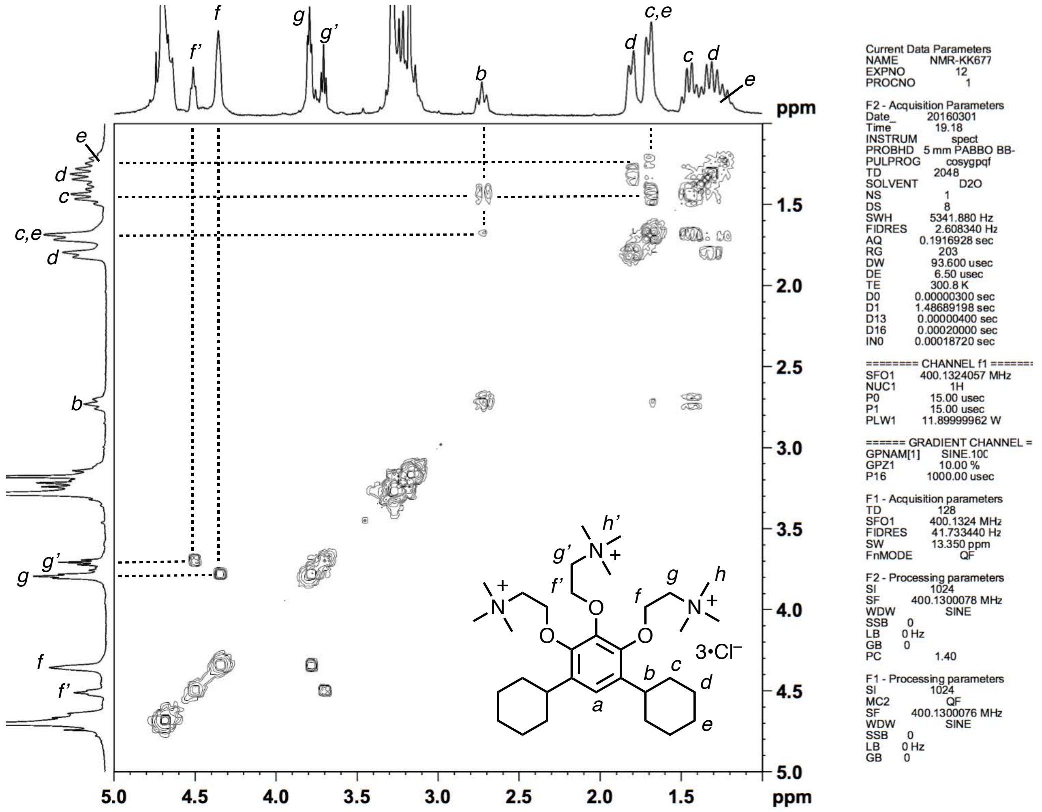

Supplementary Fig. 8 ^{1}H-^{1}H COSY spectrum (400 MHz, D_2O, ~2 mM, room temperature) of **CHA**.

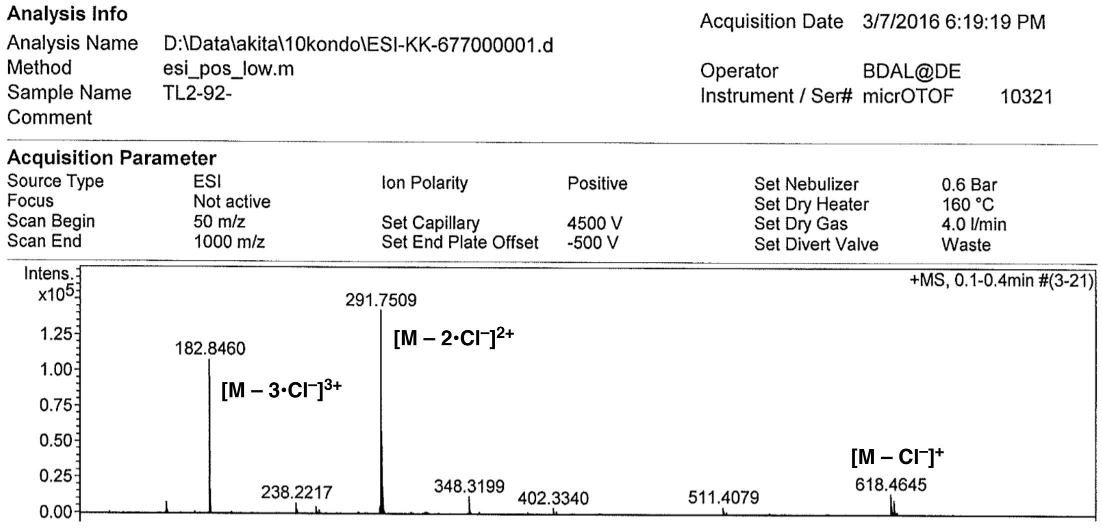

Supplementary Fig. 9 ESI-TOF MS spectrum (CH_3OH, room temperature) of **CHA**.

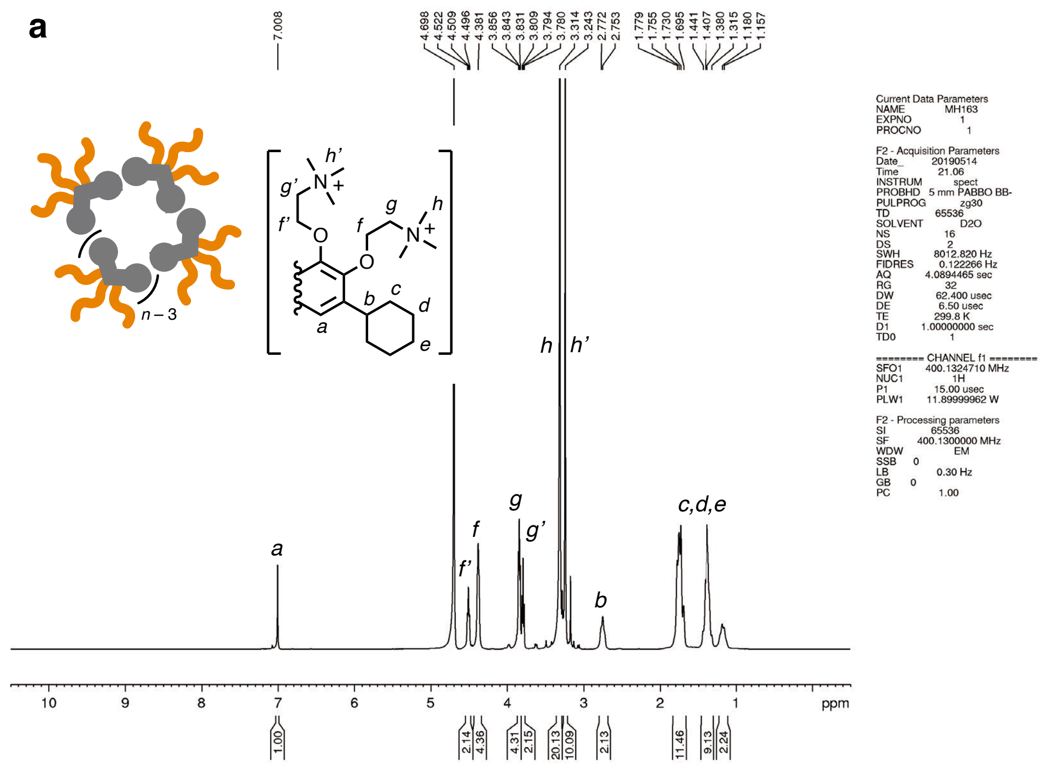

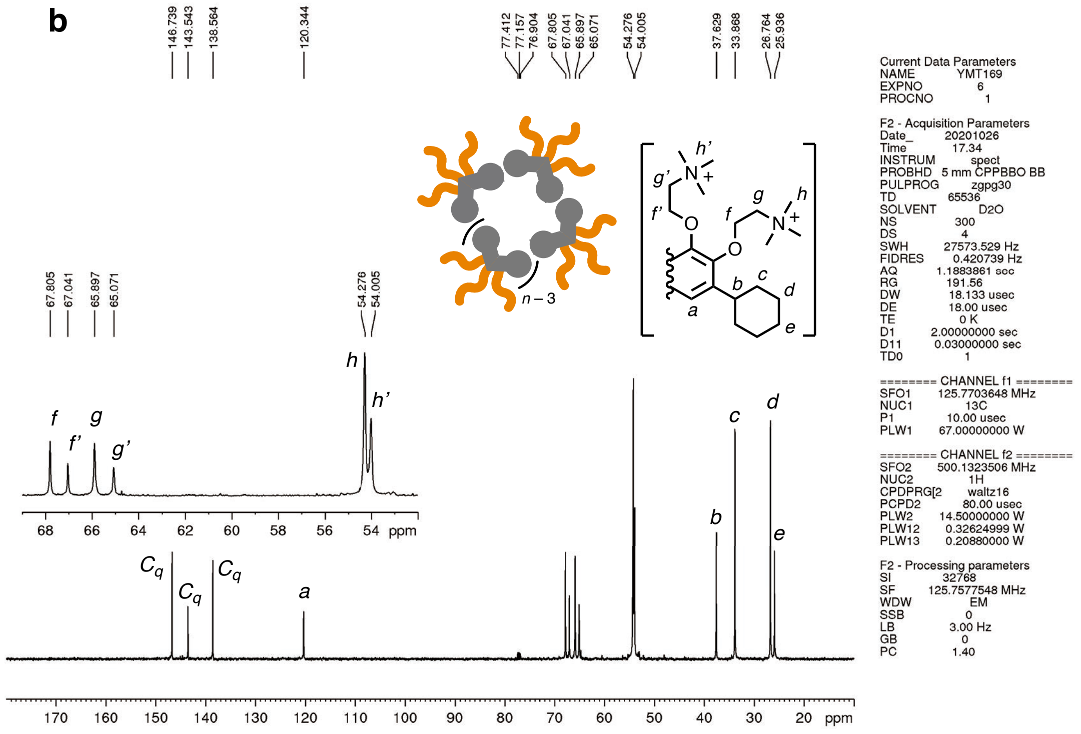

Supplementary Fig. 10 (a) ^{1}H NMR (400 MHz, room temperature) and (b) ^{13}C NMR (125 MHz, room temperature) spectra of (**CHA**)$_n$ in D_2O (170 mM based on **CHA**).

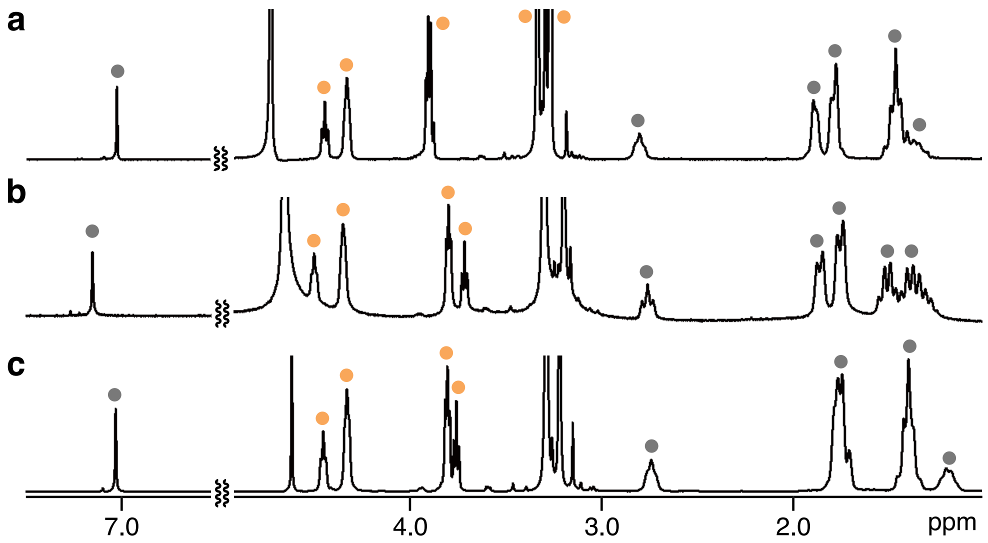

Supplementary Fig. 11 (a) ^{1}H NMR spectrum (400 MHz, room temperature) of **CHA** in CD_3OD (10 mM). Concentration-dependent ^{1}H NMR spectra (400 MHz, D_2O, room temperature, TMS as an external standard) of micelle $(\mathbf{CHA})_n$: (b) 10 mM and (c) 170 mM based on **CHA**.

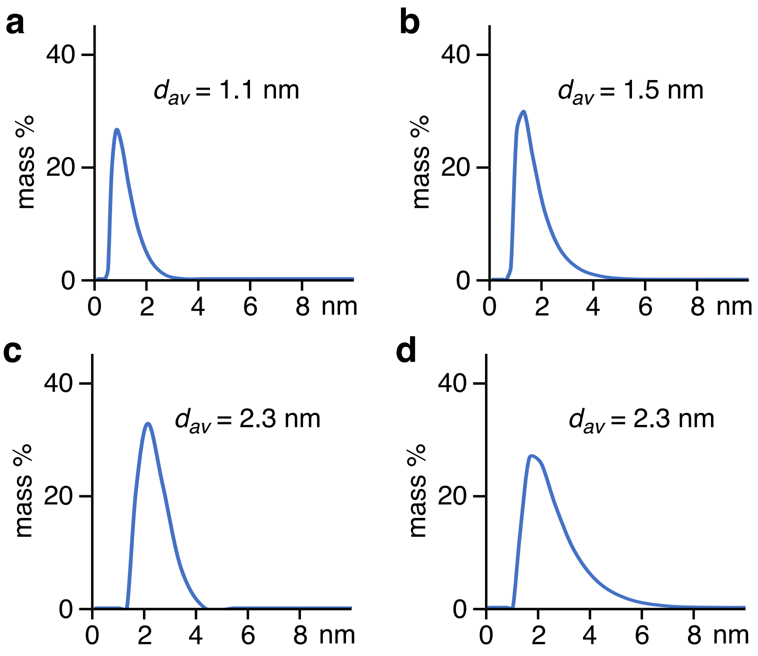

Supplementary Fig. 12 Concentration-dependent DLS charts (H_2O, room temperature) of micelle $(\mathbf{CHA})_n$: (a) 10, (b) 100, (c) 170, and (d) 300 mM based on **CHA**.

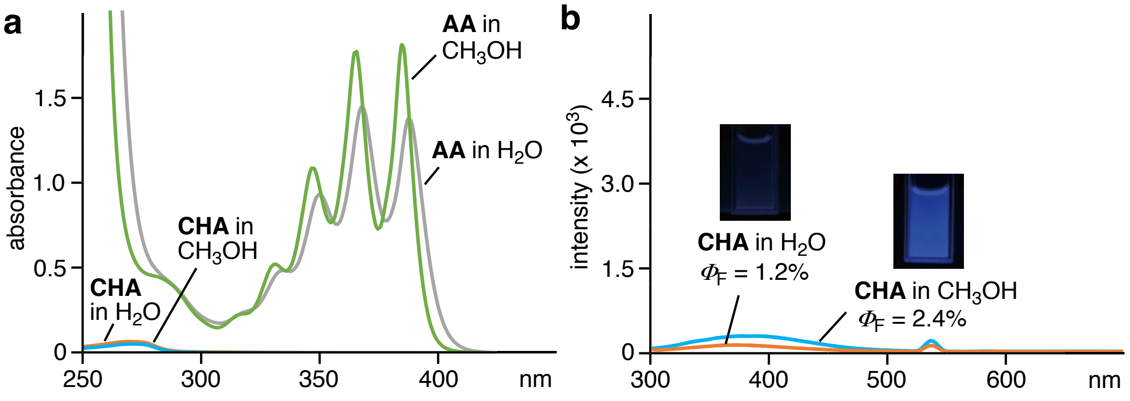

Supplementary Fig. 13 (a) UV-visible spectra (room temperature, 1.0 mM) of **CHA** and **AA**. (b) Fluorescence spectra (room temperature, λ_{ex} = 270 nm, 1.0 mM) of **CHA**.

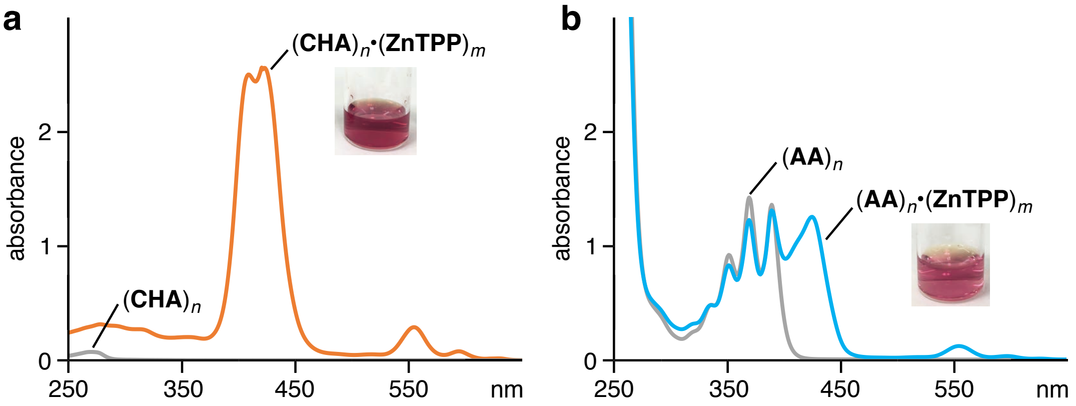

Supplementary Fig. 14 UV-visible spectra (H_2O, room temperature, 1.0 mM based on the corresponding amphiphiles) of (a) (**CHA**)$_n$•(**ZnTPP**)$_m$ and (**CHA**)$_n$, and (b) (**AA**)$_n$•(**ZnTPP**)$_m$ and (**AA**)$_n$.

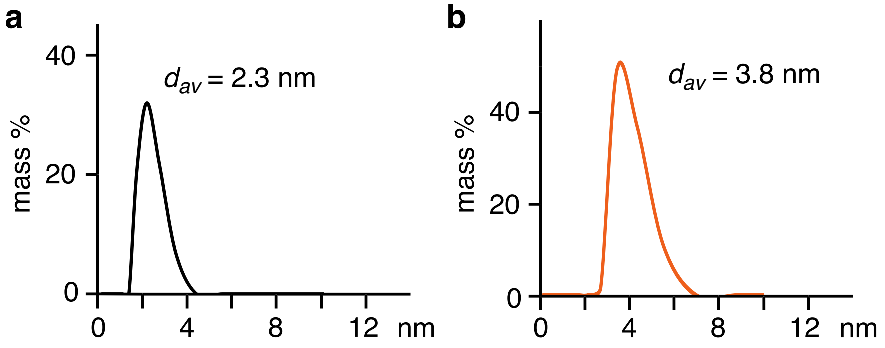

Supplementary Fig. 15 DLS charts (H_2O, room temperature) of (a) (**CHA**)$_n$ (170 mM based on **CHA**) and (b) (**CHA**)$_n$•(**ZnTPP**)$_m$ (1.0 mM based on **CHA**).

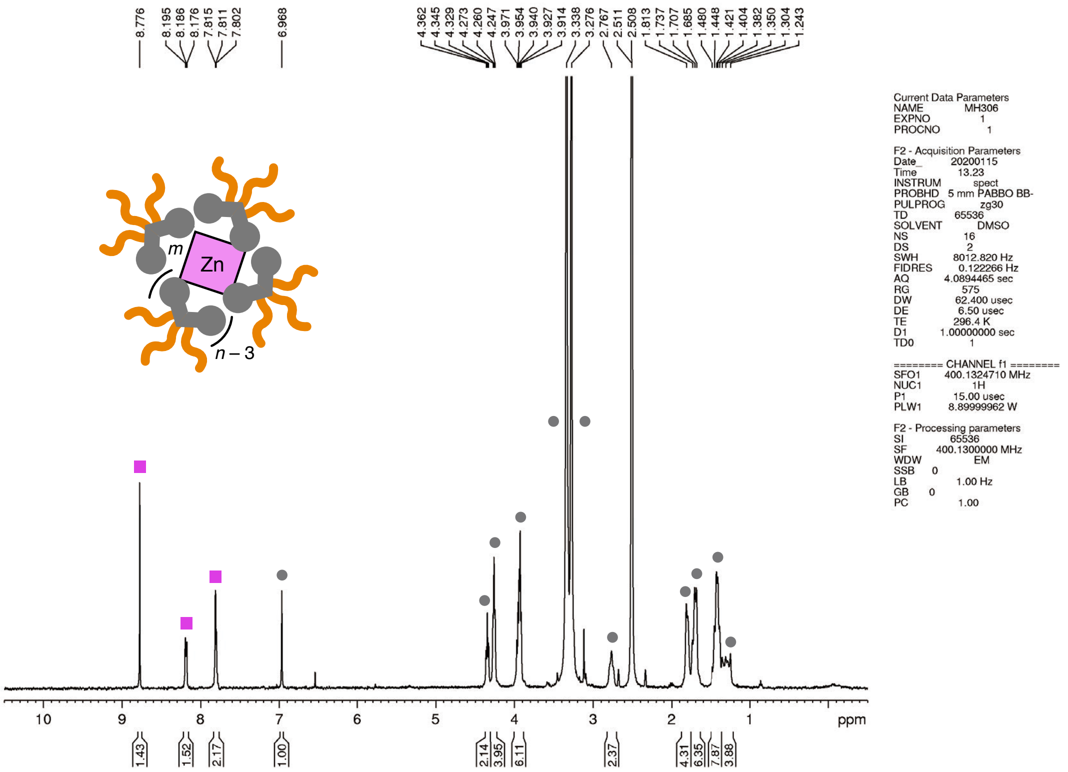

Supplementary Fig. 16 ^{1}H NMR spectrum (400 MHz, DMSO-d_6, room temperature) of isolated (**CHA**)$_n$•(**ZnTPP**)$_m$.

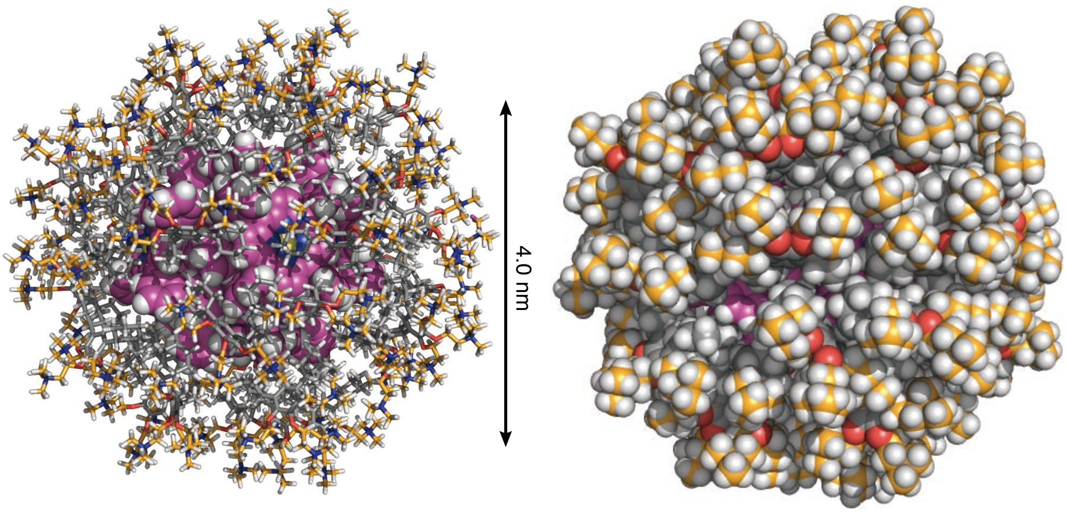

Supplementary Fig. 17 Optimized structure of (**CHA**)$_{45}$•(**ZnTPP**)$_9$ (white: hydrogen; gray, orange, and purple: carbon, red: oxygen, blue: nitrogen, yellow: zinc).

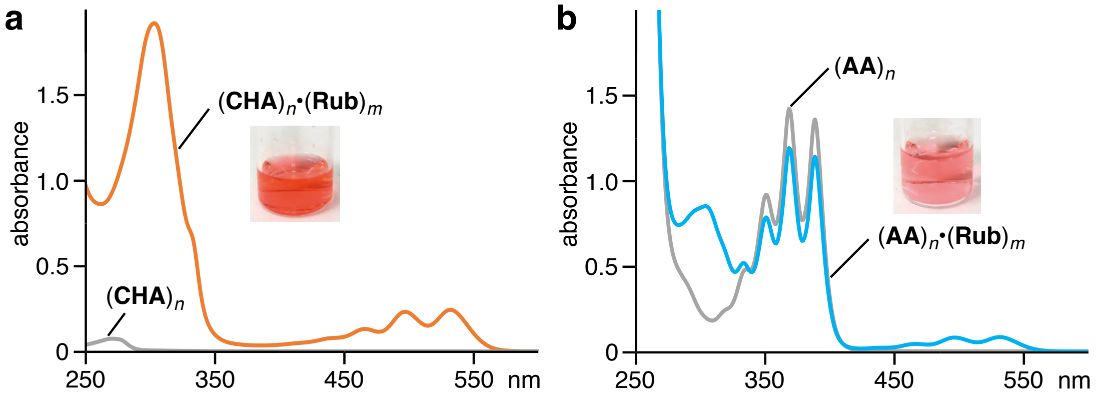

Supplementary Fig. 18 UV-visible spectra (H_2O, room temperature, 1.0 mM based on the corresponding amphiphiles) of (a) (**CHA**)$_n$•(**Rub**)$_m$ and (**CHA**)$_n$, and (b) (**AA**)$_n$•(**Rub**)$_m$ and (**AA**)$_n$.

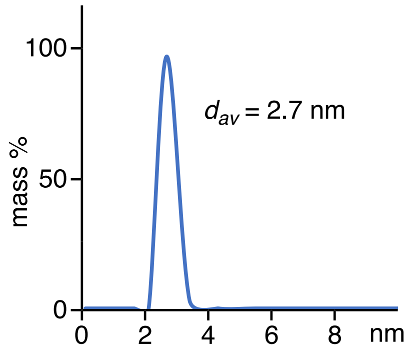

Supplementary Fig. 19 DLS chart (H_2O, room temperature, 1.0 mM based on **CHA**) of (**CHA**)$_n$•(**Rub**)$_m$.

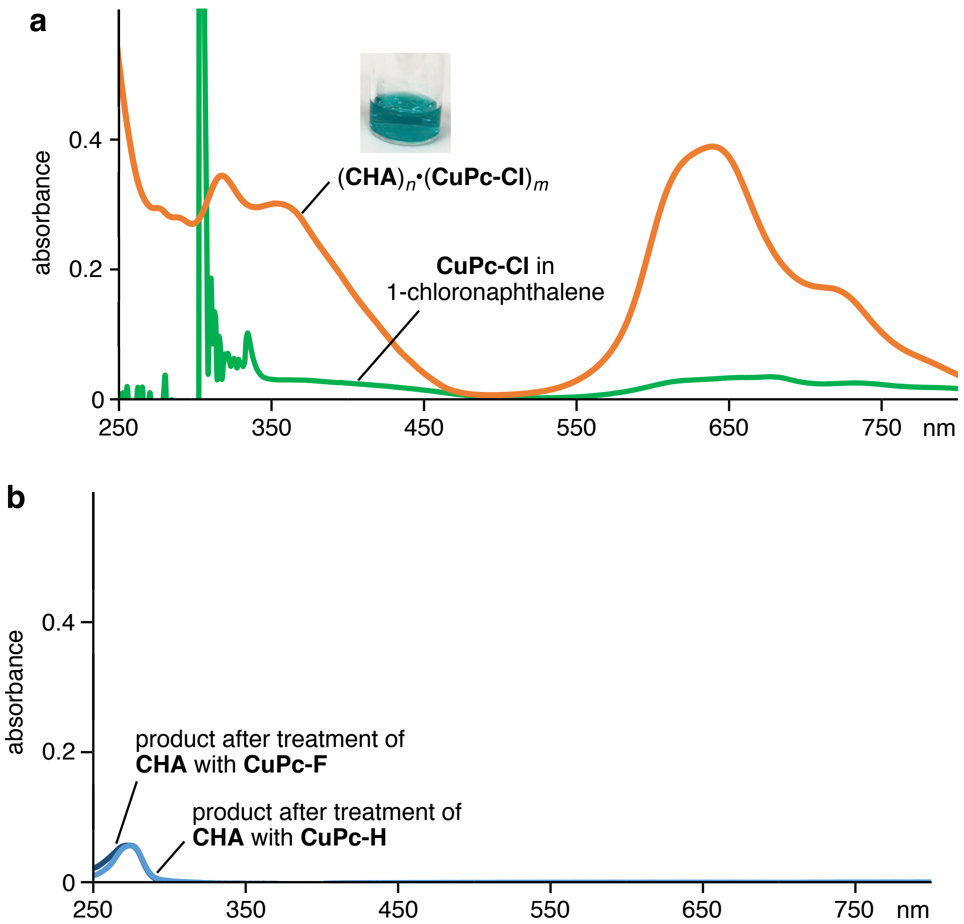

Supplementary Fig. 20 (a) UV-visible spectra (H_2O, room temperature, 1.0 mM based on **CHA**) of **(CHA)**$_n$**•(CuPc-Cl)**$_m$ and **CuPc-Cl** in 1-chloronaphthalene. (b) UV-visible spectra (H_2O, room temperature, 1.0 mM based on **CHA**) of the products after the treatment of **CHA** with **CuPc-H** or **CuPc-F**.

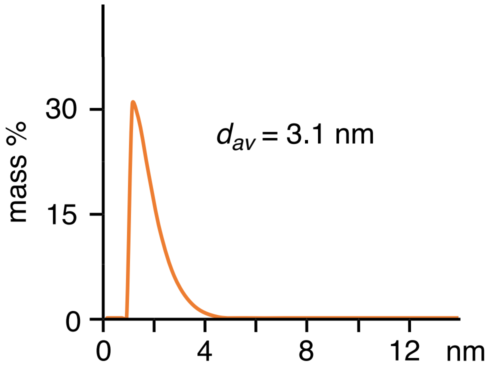

Supplementary Fig. 21 DLS chart (H_2O, room temperature, 1.0 mM based on **CHA**) of **(CHA)**$_n$**•(CuPc-Cl)**$_m$.

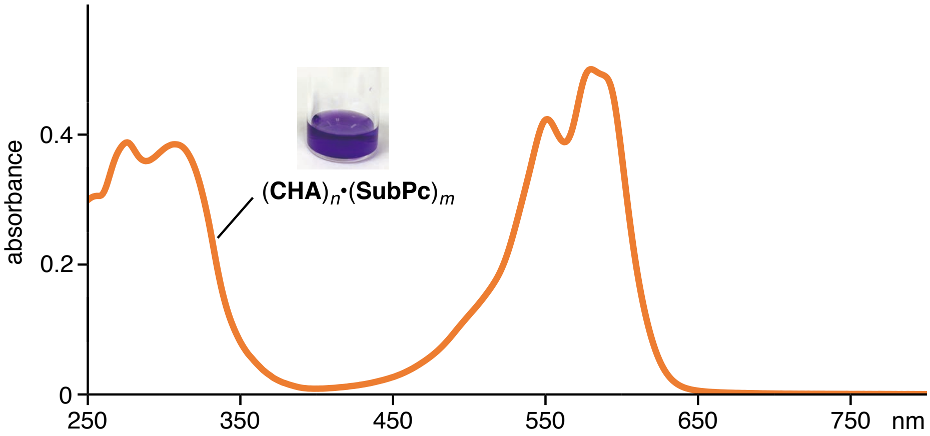

Supplementary Fig. 22 UV-visible spectrum (H_2O, room temperature, 1.0 mM based on **CHA**) of (**CHA**)$_n$•(**SubPc**)$_m$.

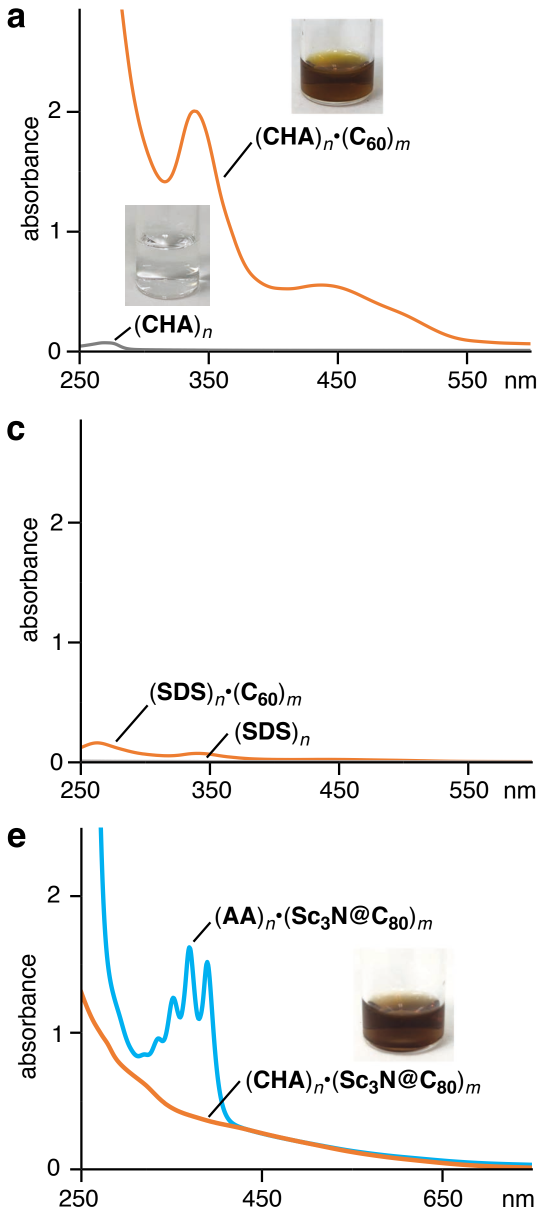

Supplementary Fig. 23 UV-visible spectra (H_2O, room temperature, 1.0 mM based on the corresponding amphiphiles) of (a) **(CHA)**$_n$**•(C$_{60}$)**$_m$ and **(CHA)**$_n$, (b) **(AA)**$_n$**•(C$_{60}$)**$_m$ and **(AA)**$_n$, (c) **(SDS)**$_n$**•(C$_{60}$)**$_m$ and **(SDS)**$_n$, (d) **(CHA)**$_n$**•(C$_{70}$)**$_m$ and **(AA)**$_n$**•(C$_{70}$)**$_m$, and (e) **(CHA)**$_n$**•(Sc$_3$N@C$_{80}$)**$_m$ and **(AA)**$_n$**•(Sc$_3$N@C$_{80}$)**$_m$.

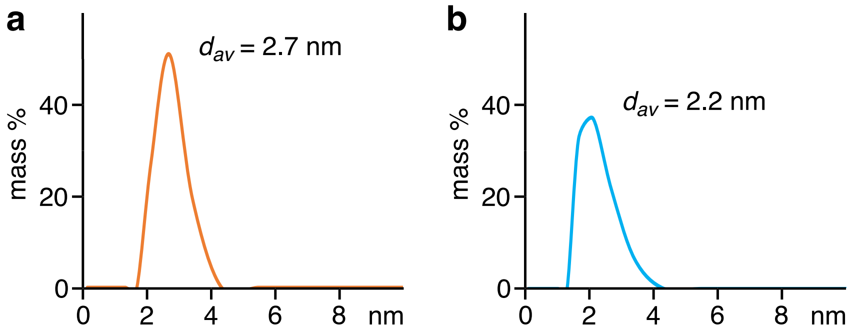

Supplementary Fig. 24 DLS charts (H_2O, room temperature, 1.0 mM based on the corresponding amphiphiles) of (a) (**CHA**)$_n$•(**C$_{60}$**)$_m$ and (b) (**AA**)$_n$•(**C$_{60}$**)$_m$.

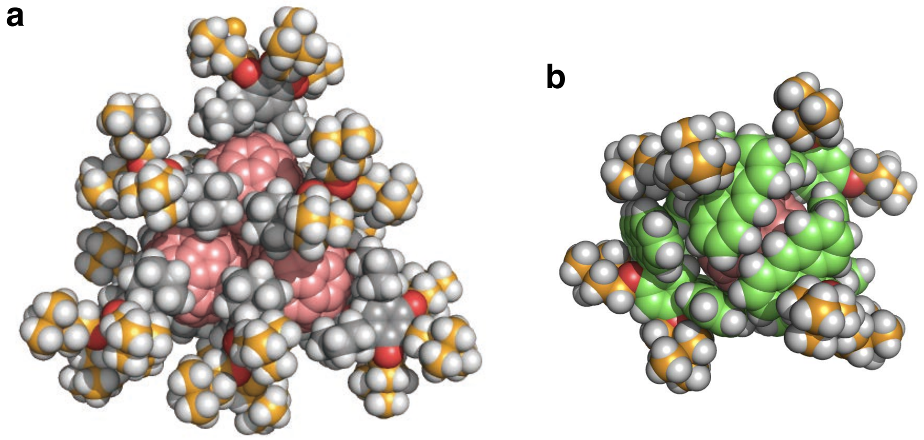

Supplementary Fig. 25 Optimized structures of (a) (**CHA**)$_{10}$•(**C$_{60}$**)$_4$ (white: hydrogen; gray, orange, and pink: carbon, red: oxygen, blue: nitrogen) and (b) (**AA**)$_5$•**C$_{60}$** (white: hydrogen; green, orange, and pink: carbon, red: oxygen, blue: nitrogen).

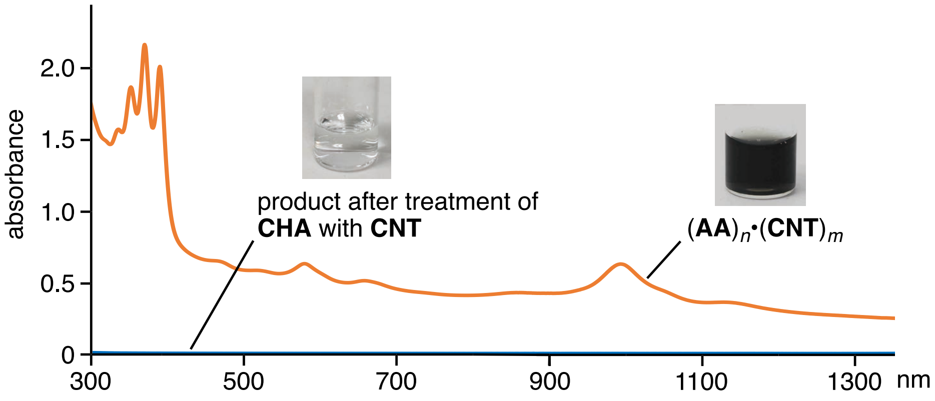

Supplementary Fig. 26 UV-visible-NIR spectra (H_2O, room temperature, 1.0 mM based on **CHA** or **AA**) of the product after treatment of **CHA** with **CNT** and (**AA**)$_n$•(**CNT**)$_m$.

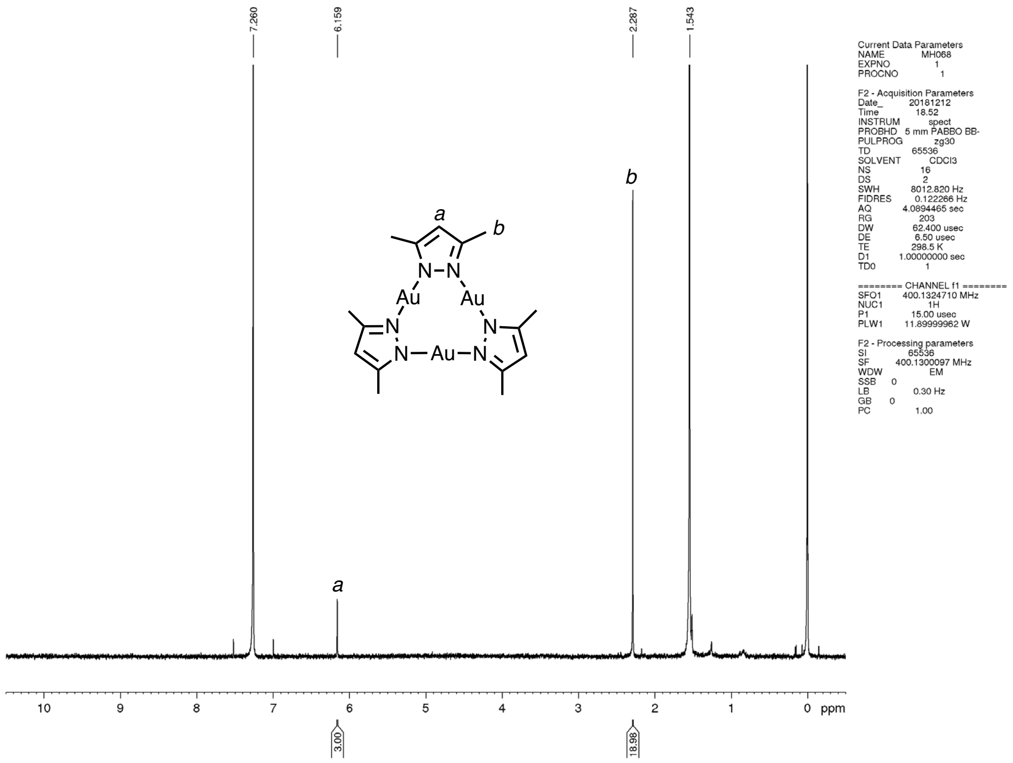

Supplementary Fig. 27 ^{1}H NMR spectrum (400 MHz, $CDCl_3$, room temperature) of **AuPz**.

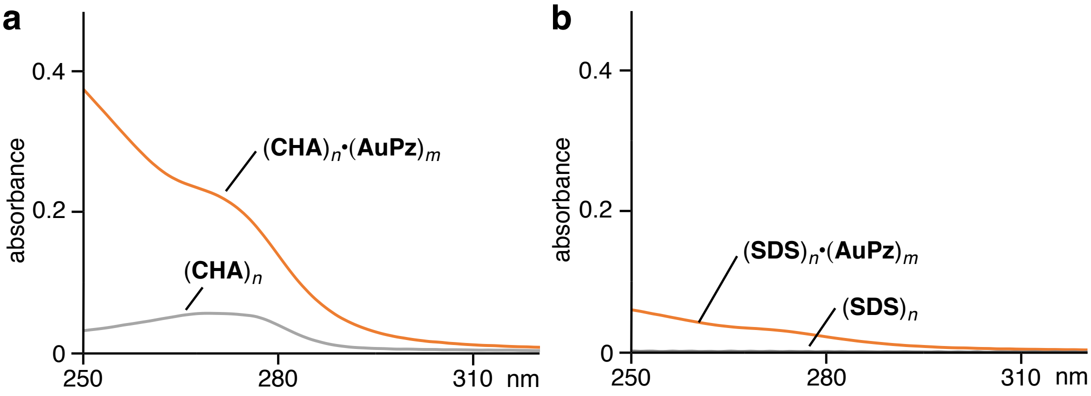

Supplementary Fig. 28 UV-visible spectra (room temperature, 1.0 mM based on **CHA** or **SDS**) of (a) (**CHA**)$_n$•(**AuPz**)$_m$ and (**CHA**)$_n$, and (b) (**SDS**)$_n$•(**AuPz**)$_m$ and (**SDS**)$_n$.

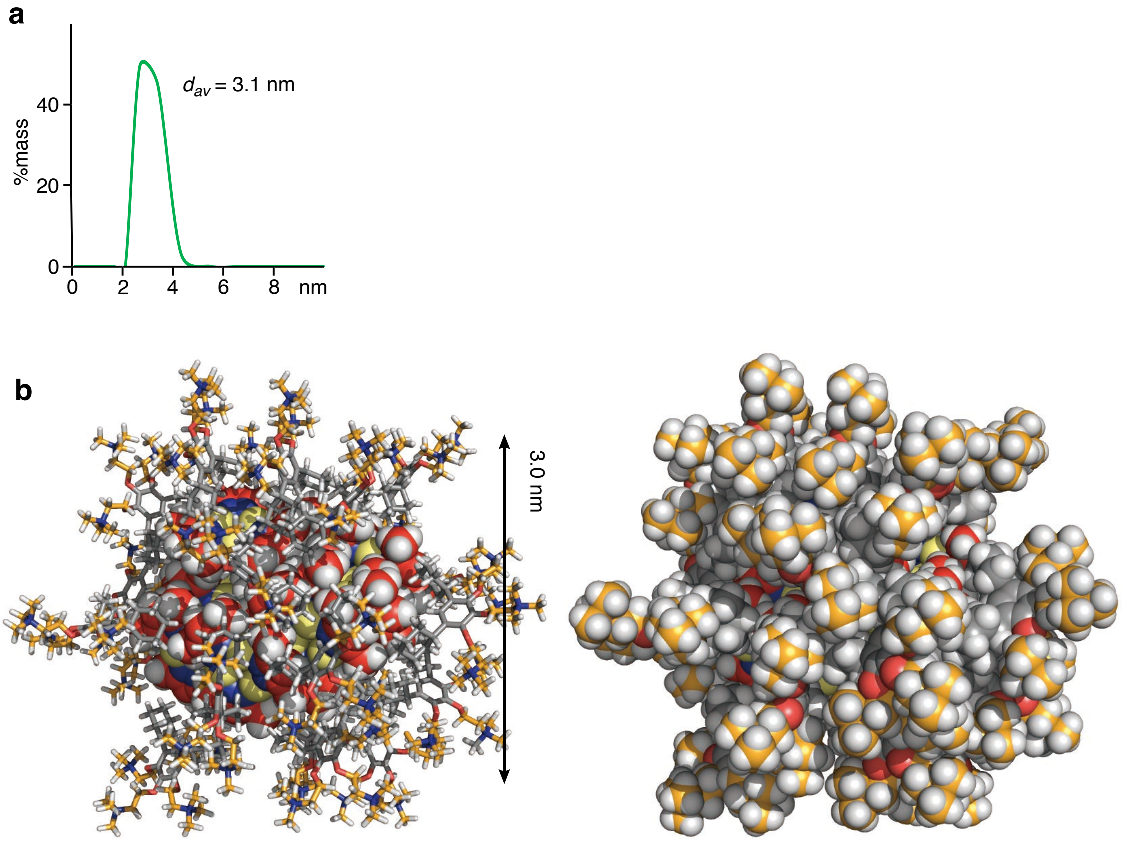

Supplementary Fig. 29 (a) DLS chart (H_2O, room temperature, 1.0 mM based on **CHA**) of a) $(\mathbf{CHA})_n$•$(\mathbf{AuPz})_m$ and (b) the optimized structure of $(\mathbf{CHA})_{22}$•$(\mathbf{AuPz})_{12}$ (white: hydrogen; gray, orange, and scarlet: carbon, red: oxygen, blue: nitrogen, yellow: gold).

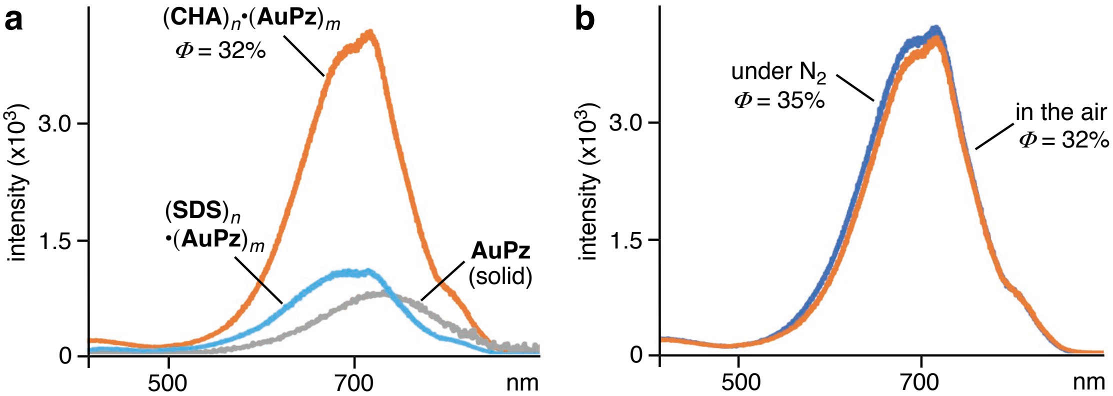

Supplementary Fig. 30 (a) Emission spectra (H_2O, room temperature, λ_{ex} = 290 nm, 1.0 mM based on **CHA** or **SDS**) of $(\mathbf{CHA})_n$•$(\mathbf{AuPz})_m$, $(\mathbf{SDS})_n$•$(\mathbf{AuPz})_m$, and **AuPz**. (b) Emission spectra (H_2O, room temperature, λ_{ex} = 290 nm, 1.0 mM based on **CHA**) of $(\mathbf{CHA})_n$•$(\mathbf{AuPz})_m$ in the air or under a N_2 atmosphere.

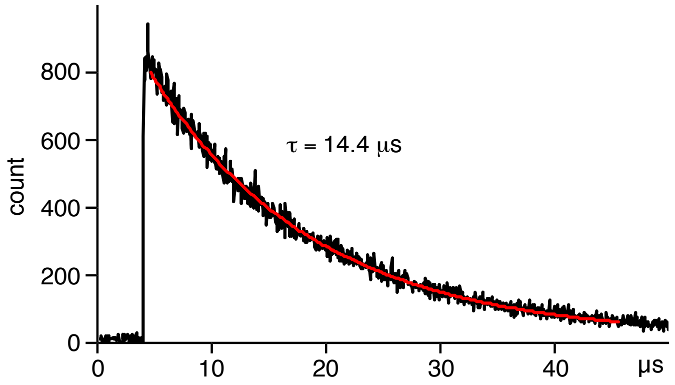

Supplementary Fig. 31 Emission decay profile (H_2O, room temperature, λ_{ex} = 280 nm, $\lambda_{det.}$ = 700 nm) of **(CHA)**$_n$**•(AuPz)**$_m$.

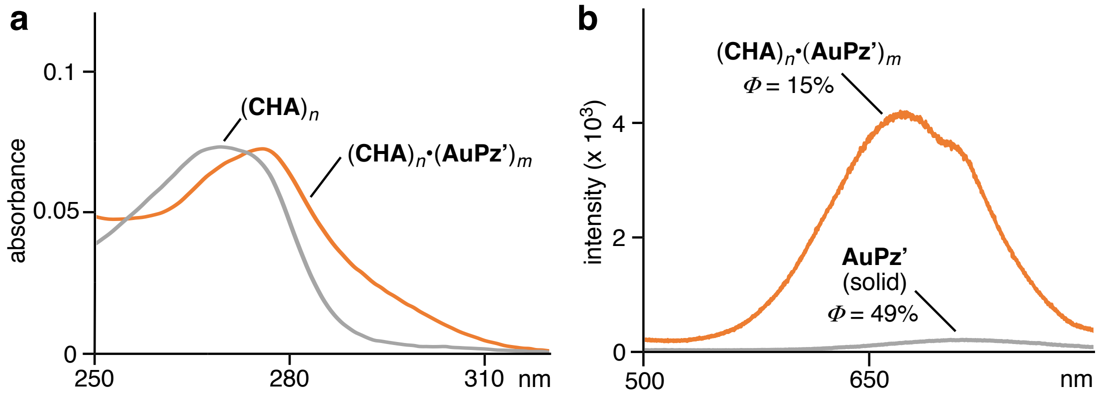

Supplementary Fig. 32 (a) UV-visible spectra (room temperature, 1.0 mM based on **CHA**) of **(CHA)**$_n$**•(AuPz')**$_m$ and **(CHA)**$_n$. (b) Emission spectra (H_2O, room temperature, λ_{ex} = 280 nm, 1.0 mM based on **CHA**) of **(CHA)**$_n$**•(AuPz')**$_m$ and **AuPz'** (solid).

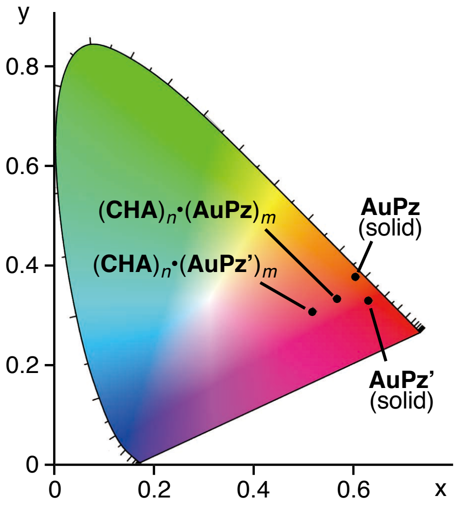

Supplementary Fig. 33 CIE coordinate diagram (H_2O, room temperature) of **(CHA)**$_n$**•(AuPz)**$_m$, **(CHA)**$_n$**•(AuPz')**$_m$, **AuPz**, and **AuPz'**.

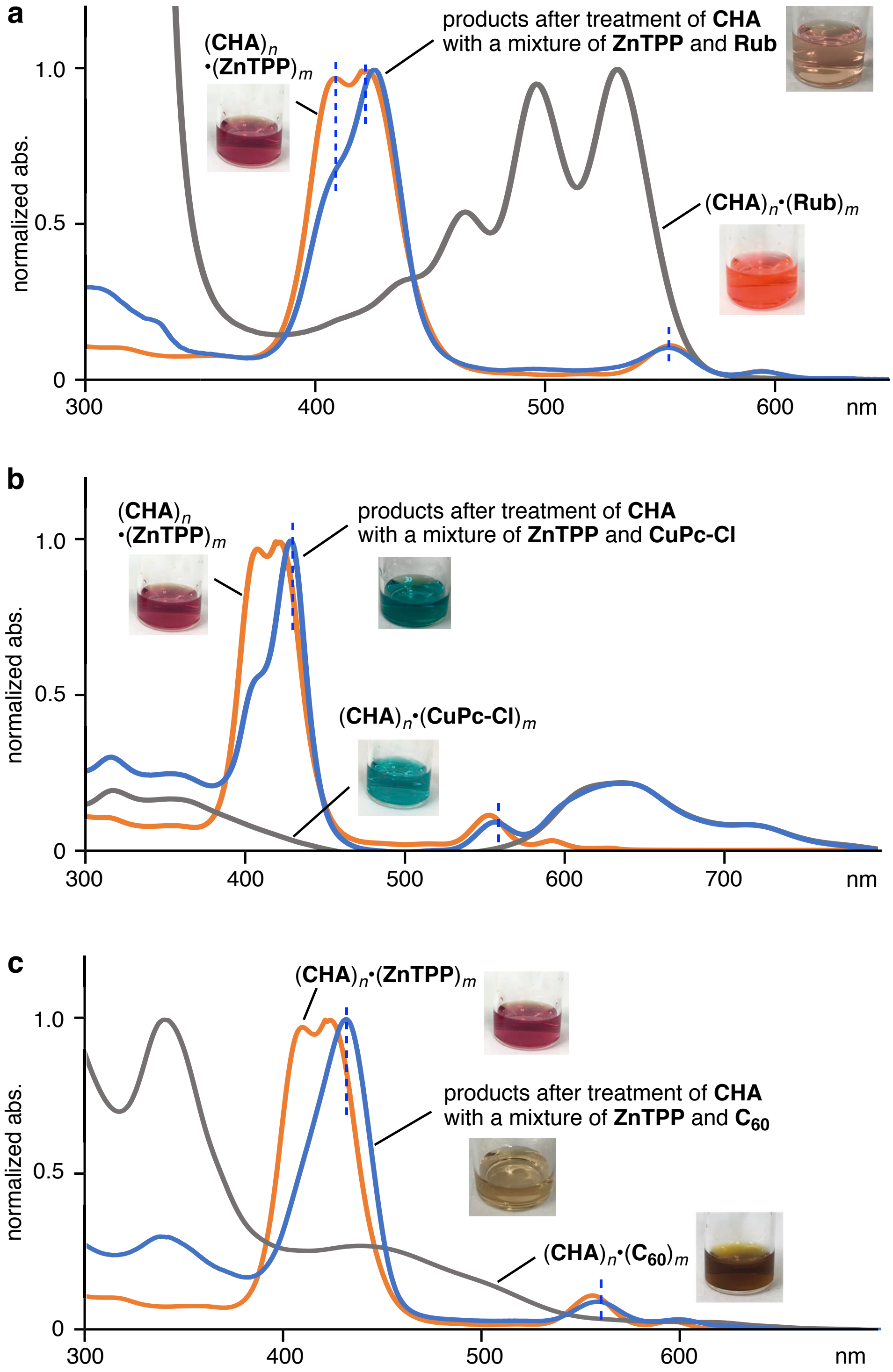
a
b
c
normalized abs.
1.0
0.5
0
300
400
500
600
700
nm
(CHA)n •(ZnTPP)m
products after treatment of CHA with a mixture of ZnTPP and Rub
(CHA)n•(Rub)m
products after treatment of CHA with a mixture of ZnTPP and CuPc-Cl
(CHA)n•(CuPc-Cl)m
(CHA)n•(ZnTPP)m
products after treatment of CHA with a mixture of ZnTPP and C60
(CHA)n•(C60)m

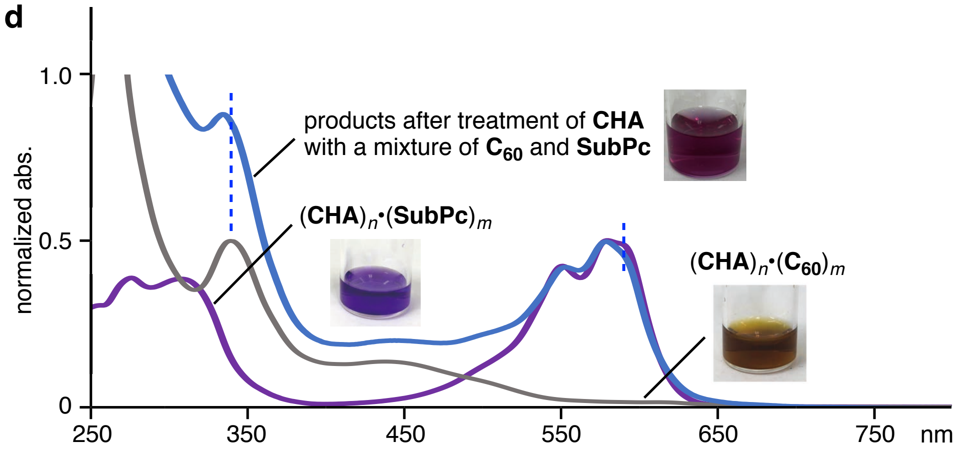

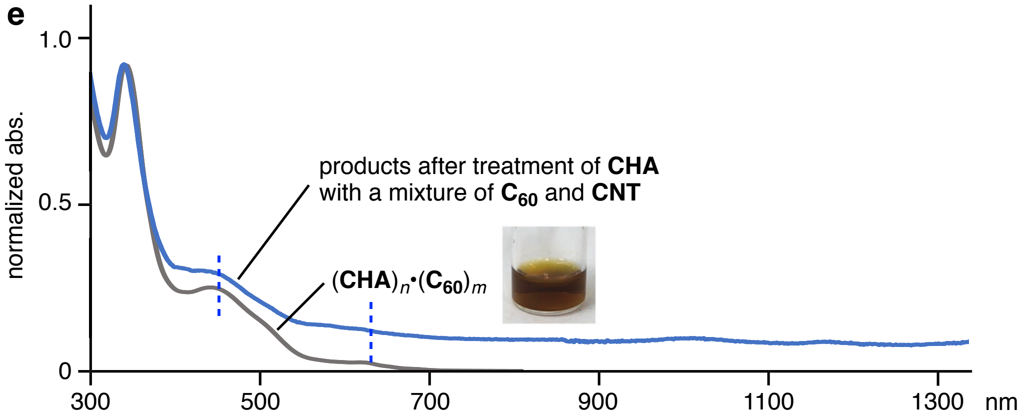

Supplementary Fig. 34 UV-visible(-NIR) spectra (H_2O, room temperature, 1.0 mM based on **CHA**) of products after treatment of **CHA** with a mixture of (a) **ZnTPP** and **Rub**, (b) **ZnTPP** and **CuPc-Cl**, (c) **ZnTPP** and **C_{60}**, (d) **C_{60}** and **SubPc**, and (e) **C_{60}** and **CNT**.

Supplementary Methods

Formation of (CHA)$_n$•(Rub)$_m$

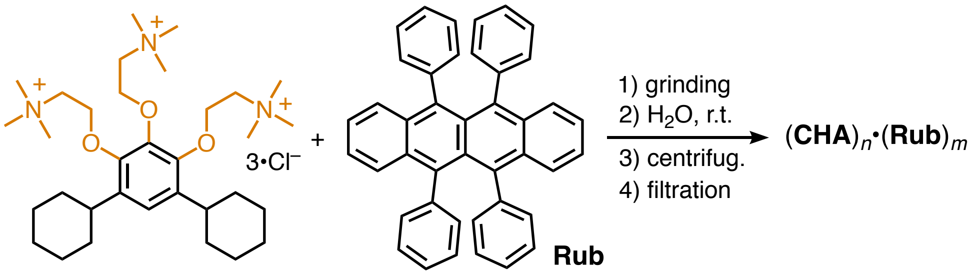

A mixture of **CHA** (1.4 mg, 2.1 μmol) and rubrene (**Rub**; 1.1 mg, 2.1 μmol) was ground for 6 min using an agate mortar and pestle. After the addition of H_2O (2.1 ml) to the mixture, the suspended solution was centrifuged (16,000 g, 10 min) and then filtered by a membrane filter (pore size: 200 nm) to give a clear red solution of (**CHA**)$_n$•(**Rub**)$_m$. The structure of (**CHA**)$_n$•(**Rub**)$_m$ was confirmed by UV-visible and DLS analyses, and the concentration of encapsulated **Rub** (0.2 mM) was estimated by UV-visible analysis. The same procedure using **AA** (1.4 mg, 2.0 μmol) and **Rub** (0.6 mg, 1.9 μmol) or **SDS** (0.6 mg, 2.0 μmol) and **Rub** (0.6 mg, 2.1 μmol) afforded a clear pale red solution of (**AA**)$_n$•(**Rub**)$_m$ or (**SDS**)$_n$•(**Rub**)$_m$.

Formation of (CHA)$_n$•(SubPc)$_m$

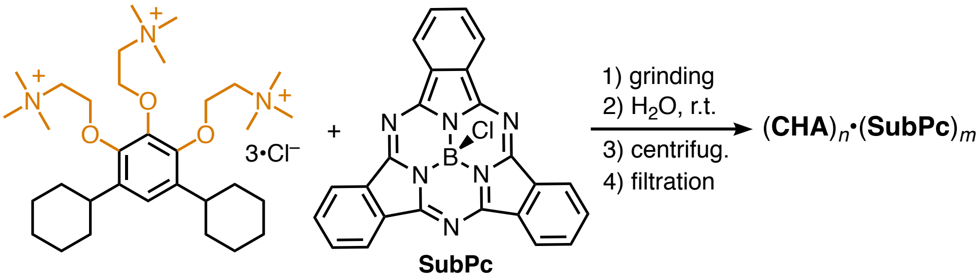

A mixture of **CHA** (1.3 mg, 2.0 μmol) and subphthalocyanine (**SubPc**; 0.4 mg, 1.0 μmol) was ground for 6 min using an agate mortar and pestle. After the addition of H_2O (2.0 ml) to the mixture, the suspended solution was centrifuged (16,000 g, 10 min) and then filtered by a membrane filter (pore size: 200 nm) to give a clear purple solution of (**CHA**)$_n$•(**SubPc**)$_m$. The formation of (**CHA**)$_n$•(**SubPc**)$_m$ was confirmed by UV-visible analysis.

Synthesis of AA

A dry THF solution (35 ml) of 1,5-dibromo-2,4-dimethoxybenene (0.692 g, 2.34 mmol), $PdCl_2(PhCN)_2$ (44.4 mg, 0.116 mmol), and $P(t\text{-}Bu)_3$•HBF_4 (67.5 mg) was added to a dry THF solution (15 ml) of 9-anthrylzinc chloride, prepared from 9-bromoanthracene (1.51 g, 5.86 mmol) and a hexane solution (1.6 M) of *n*-butyllithium (4.1 ml, 6.5 mmol) under N_2. The mixture was stirred at 85 ºC for 2 d. The precipitated product was collected and washed with CH_3OH, hexane, and $CHCl_3$ to afford 1,5-di(9-anthryl)-2,4-dimethoxybenzene (0.635 g, 1.29 mmol; 55% yield) as a pale yellow solid. A CH_2Cl_2 solution (1.0 M) of BBr_3 (9.2 ml, 9.2 mmol) was added dropwise to a dry CH_2Cl_2 (20 ml) of 1,5-di(9-anthryl)-2,4-dimethoxybenzene (1.50 g, 3.06 mmol) at 0 ºC and then the mixture was stirred at 50 ºC for 3 h under N_2. The reaction was quenched with H_2O and the organic solvent was removed under vacuum. The obtained solid was washed with H_2O to afford 1,5-di(9-anthryl)-2,4-dihydroxybenzene (1.25 g, 2.70 mmol; 88% yield) as a white solid[1,2].

When 1,5-di(9-anthryl)-2,4-dihydroxybenzene (0.50 g, 1.08 mmol) and NaOH (2.25 g, 56.2 mmol) were stirred in toluene (40 ml) at 80 ºC for 30 min under N_2, 2-chloro-*N,N*-dimethylethanamine hydrochloride (1.24 g, 8.64 mmol) was added to the solution. The resultant mixture was further stirred at 130 ºC for 12 h. The crude product was extracted with EtOAc and washed with hexane to afford a white solid (0.419 g, 0.692 mmol; 64% yield). The product (0.347 g, 0.574 mmol) and methyl iodide (0.18 ml, 2.9 mmol) were stirred in CH_3CN (10 ml) overnight at room temperature. The solvent was removed under vacuum and washed with acetone and hexane to afford a white solid (0.320 g, 0.360 mmol; 63% yield). The obtained white solid (0.251 g, 0.282 mmol) and AgCl (0.121 mg, 0.844 mmol) were stirred in H_2O (4 ml) at 80 ºC for 22 h. After the addition of CH_3OH (20 ml), the resultant solution was filtrated and concentrated under vacuum. The obtained solid was washed with $CHCl_3$ and hexane to afford **AA** (143 mg, 0.203 mmol; 72% yield) as a white solid[1,2].

Compound **AA**: ^1H NMR (400 MHz, CD_3OD, r.t.): δ 2.45 (s, 18H), 3.44 (t, 4H, J = 4.4 Hz), 4.58 (br, 4H), 7.33 (s, 1H), 7.41 (s, 1H), 7.51-7.54 (m, 8H), 7.89-7.91 (m, 4H), 8.10-8.13 (m, 4H), 8.60 (s, 2H). ESI-TOF MS (CH_3OH): *m/z* Calcd. 669.3, Found 669.3 $[M – Cl^-]^+$; Calcd. 317.2, Found 317.2 $[M – 2•Cl^-]^{2+}$.

Synthesis of Chloro(tetrahydrothiophene)Au(I)

When tetrahydrothiophene (0.50 ml, 5.67 mmol) was added dropwise to an EtOH solution (16 ml) of $HAuCl_4{\bullet}4H_2O$ (1.07 g, 2.60 mmol) at room temperature, a suspended white solution was formed for 3 h. The crude product was collected and washed with CH_3OH to afford chloro(tetrahydrothiophene)Au(I) (0.649 g, 2.02 mmol; 78% yield) as a white solid[3].

Synthesis of AuPz

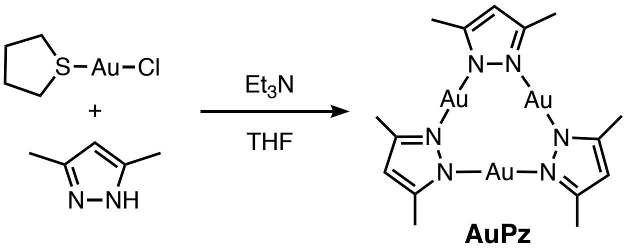

Chloro(tetrahydrothiophene)Au(I) (69.5 mg, 0.217 mmol), 3,5-dimethylpyrazole (50.9 mg, 0.231 mmol), triethylamine (33 μl, 0.24 mmol), dry and degassed THF (20 ml) were added to a 2-necked 50 ml glass flask filled with N_2. The resultant mixture was stirred at room temperature for 22 h. The crude product was washed with CH_3OH to afford **AuPz** (39.6 mg, 45.2 mmol; 63% yield) as a white solid[4].

^1H NMR (400 MHz, $CDCl_3$, room temperature): δ 6.16 (s, 3H), 2.29 (s, 18H). MALDI-TOF MS (dithranol): *m/z* Calcd. for $C_{15}H_{22}Au_3N_6$ $[M + H]^+$ 877.09, Found 877.10.

Synthesis of AuPz'

Chloro(tetrahydrothiophene)Au(I) (97.9 mg, 0.305 mmol), pyrazole (29.2 mg, 0.429 mmol), and triethylamine (100 μl, 0.72 mmol) were stirred in dry and degassed THF (12 ml) at room temperature for 2 d. The crude product was washed with CH_3OH to afford **AuPz'** (57.8 mg, 73.0 μmol; 72% yield) as a white solid[4].

MALDI-TOF MS (dithranol): *m/z* Calcd. for $C_9H_9Au_3N_6$ $[M + H]^+$ 793.00, Found 793.21.

Uptake studies of (CHA)$_n$ from mixtures of two different guests

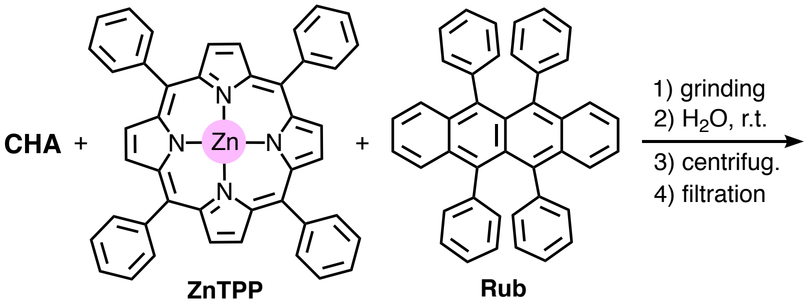

A mixture of **CHA** (1.5 mg, 2.3 μmol), **ZnTPP** (0.3 mg, 0.5 μmol), and **Rub** (0.3 mg, 0.5 μmol) was ground for 6 min using an agate mortar and pestle. After the addition of H_2O (2.0 ml) to the mixture, the suspended solution was centrifuged (16,000 g, 10 min) and then filtered by a membrane filter (pore size: 200 nm) to give a clear solution. The product structures were analyzed by UV-visible spectroscopy. The treatment of **CHA** with **ZnTPP/CuPc-Cl**, **ZnTPP/C$_{60}$**, **C$_{60}$/SubPc**, and **C$_{60}$/CNT** was examined in the same way (See Supplementary Fig. 34).

Supplementary References

1. Kondo, K., Suzuki, A., Akita, M., Yoshizawa M. Micelle-like molecular capsules with anthracene shells as photoactive hosts. *Angew. Chem. Int. Ed.* **52**, 2308–2312 (2013).
2. Kondo, K., Akita, M., Nakagawa, T., Matsuo, Y., Yoshizawa, M. A V-shaped polyaromatic amphiphile: solubilization of various nanocarbons in water and enhanced photostability. *Chem. Eur. J.* **21**, 12741–12746 (2015).
3. Uson, R., Laguna, A. Laguna, M., Briggs, D. A., Murray, H. H., Fackler Jr., J. P. (Tetrahydrothiophene)gold(I) or gold(III) complexes. *Inorg. Synth.* **26**, 85–91 (1898).
4. Yang, G., Raptis, R. G. Supramolecular assembly of trimeric gold(I) pyrazolates through aurophilic attractions. *Inorg. Chem.* **42**, 261–263 (2003).
